# LRP1B: A Giant Lost in Cancer Translation

**DOI:** 10.3390/ph14090836

**Published:** 2021-08-24

**Authors:** Catarina Príncipe, Isabel J. Dionísio de Sousa, Hugo Prazeres, Paula Soares, Raquel T. Lima

**Affiliations:** 1 i3S—Instituto de Investigação e Inovação em Saúde, Universidade do Porto, 4200-135 Porto, Portugal; catarina.principe.f.santos@gmail.com (C.P.); hprazeres@i3s.up.pt (H.P.); psoares@ipatimup.pt (P.S.); 2 Cancer Signalling and Metabolism Group, IPATIMUP—Institute of Molecular Pathology and Immunology, University of Porto, 4200-135 Porto, Portugal; 3 Department of Oncology, Centro Hospitalar Universitário de São João, 4200-450 Porto, Portugal; isajose8@gmail.com; 4 Faculty of Medicine, University of Porto, 4200-319 Porto, Portugal; 5 IPO-Coimbra, Portuguese Oncology Institute of Coimbra, 3000-075 Coimbra, Portugal; 6 Department of Pathology, Faculty of Medicine, University of Porto, 4200-319 Porto, Portugal

**Keywords:** LRP1B, endocytic receptor, cancer, tumor suppressor, biomarker, prognosis, predictor of response, immune checkpoint inhibitors

## Abstract

Low-density lipoprotein receptor-related protein 1B (LRP1B) is a giant member of the LDLR protein family, which includes several structurally homologous cell surface receptors with a wide range of biological functions from cargo transport to cell signaling. LRP1B is among the most altered genes in human cancer overall. Found frequently inactivated by several genetic and epigenetic mechanisms, it has mostly been regarded as a putative tumor suppressor. Still, limitations in LRP1B studies exist, in particular associated with its huge size. Therefore, LRP1B expression and function in cancer remains to be fully unveiled. This review addresses the current understanding of LRP1B and the studies that shed a light on the LRP1B structure and ligands. It goes further in presenting increasing knowledge brought by technical and methodological advances that allow to better manipulate LRP1B expression in cells and to more thoroughly explore its expression and mutation status. New evidence is pushing towards the increased relevance of LRP1B in cancer as a potential target or translational prognosis and response to therapy biomarker.

## 1. Low-Density Lipoprotein (LDL) Receptor (LDRL)-Related Protein 1B (LRP1B)

### 1.1. A Giant Member from a Large Receptor Family

**Figure 1:**
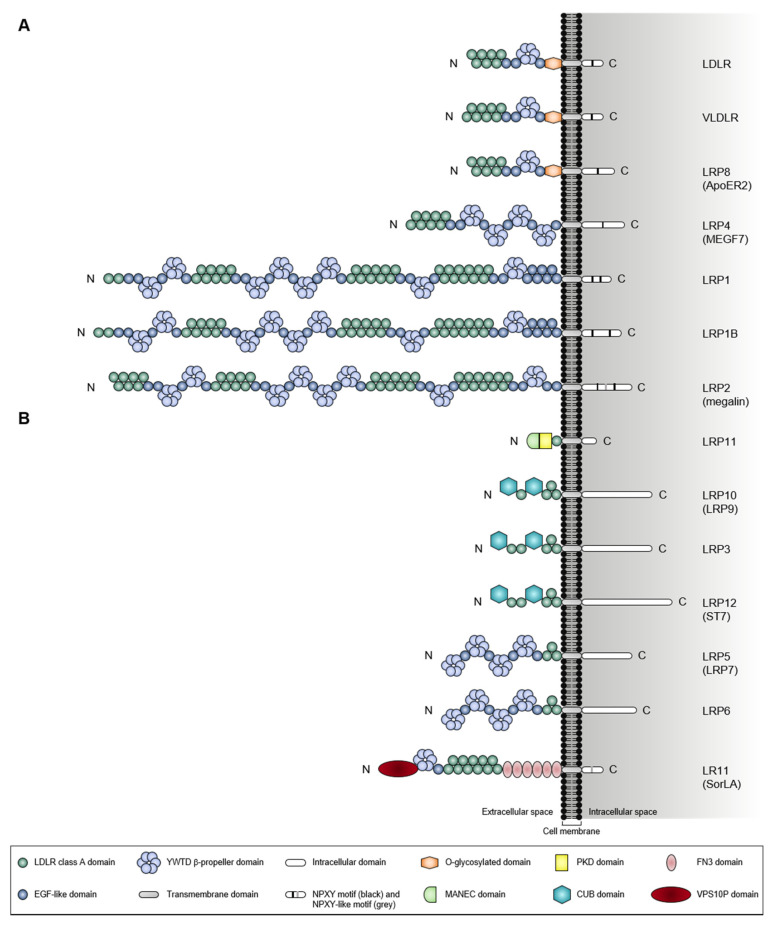
**Human LDLR family members**. Schematic representation depicting the domain organization of core (**A**) and distant-related (**B**) members of the human LDLR family. All members are anchored to the cell membrane by a single membrane-spanning domain and contain an intracellular domain ranging from 50 to 346 amino acids. These are type I membrane proteins (i.e., extracellular N-terminus and intracellular C-terminus; the presence of an N-terminus signal peptide). The core members (**A**) contain an extracellular domain built from a minimal central unit of an N-terminus cluster of LDLRA domains followed by a C-terminus cluster of EGF-like and YWTD β-propeller domains. LDLR, VLDLR, and LRP8 contain an additional extracellular O-glycosylated domain adjacent to the transmembrane domain. The core members also have an intracellular domain with at least one NPXY motif. The distant-related members (**B**) contain at least one of the extracellular domains of the core members. In addition, these also include domains that are not present in the core members. Abbreviations: LDLR, low-density lipoprotein receptor; VLDLR, very low-density lipoprotein re-ceptor; LRP, low-density lipoprotein receptor-related protein; EGF, epidermal growth fac-tor, YWTD, tyrosine-tryptophan-threonine-aspartate; NPXY, aspara-gine-proline-x-tyrosine (where x is any amino acid); MANEC, motif at the N-terminus with eight cysteines; PKD, polycystic kidney disease; CUB, complement C1r/C1s, Uegf, Bmp1; FN3, fibronectin type-III; VPS10P, vacuolar protein sorting ten protein.

The low-density lipoprotein (LDL) receptor (LDRL)-related protein 1B (LRP1B) belongs to the LDLR protein family [1,2,3], a large class of cell-surface proteins with diverse functions, ranging from receptor-mediated endocytosis to cellular signaling (extensively reviewed in [4,5,6]). In humans, the LDLR family is comprised of seven core members and seven distant-related members (Figure 1). LDLR core members include LDLR itself, VLDLR, LRP1, LRP2, LRP4, LRP8, and LRP1B, which share five structurally and functionally distinct domains [4,5,6]: (i) LDLR class A (LDLRA) domain, an approximately forty-amino acid sequence with six conserved cysteines, and a highly conserved cluster of negatively-charged amino acids between the fourth and sixth cysteine; (ii) epidermal growth factor (EGF)-like domain, a thirty to forty-amino acid sequence also with six conserved cysteines; (iii) tyrosine-tryptophan-threonine-aspartate (YWTD) β-propeller domain, with six contiguous YWTD or LDLR class B (LDLRB) repeats (each containing a conserved YWTD motif), that together fold into six-bladed β-propeller; (iv) transmembrane domain, and (v) intracellular domain (endodomain), with one or more conserved asparagine-proline-x-tyrosine (NPXY, where X designates any amino acid) motifs. Their extracellular domains (ectodomains) are built from a minimal central unit of an N-terminus cluster of LDLRA domains (responsible for ligand binding) followed by a C-terminus cluster of EGF-like and YWTD β-propeller domains (responsible for pH-dependent ligand release) [7,8,9]. The number of each domain (and its clusters) greatly varies among the core members. Some, but not all, receptors contain an additional extracellular O-linked sugar domain adjacent to the transmembrane domain [10]. Comparing with the extracellular domains, the intracellular domains are less conserved between the receptors, except for the NPXY motif [10].

Besides the core members, the LDLR family also includes the distant-related members LR11, LRP3, LRP5, LRP6, LRP10, LRP11, and LRP12, which are structurally and functionally highly diversified. Similar to the core members, LRP5 and LRP6 contain all three extracellular domains but with a distinct arrangement (such as the cluster of EGF-like and YWTD β-propeller domains preceding the cluster of LDLRA domains) and both lacking intracellular NPXY motifs [11]. LR11 has a vacuolar protein sorting ten protein (VPS10P) domain, in addition to the typical LDLR extracellular domains, as well as a cluster of fibronectin type-III (FN3) domains [12] and one NPXY-like motif (FANSHY, phenylalanine-alanine-asparagine-serine-histidine-tyrosine) within its intracellular domain [13]. LRP3, LRP10, LRP11, and LRP12 only have the LDLRA domain in common with the core members.

**Figure 2:**
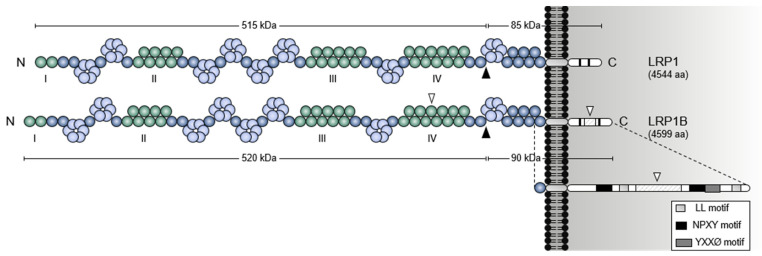
**Domain organization comparison between human LRP1 and LRP1B.** The four extracellular ligand-binding domains (I to IV) are shown. The two structural differences between LRP1 and LRP1B are indicated with white arrowheads: (i) an extra LDLRA domain in the fourth ligand-binding domain of LRP1B and (ii) a unique 33 amino acid sequence between the two NPXY motifs in the intracellular domain of LRP1B. The locations of the furin cleavage sites in the receptors are pointed out by black arrowheads. The intracellular domain of LRP1B is shown in more detail. Abbreviations: LL, dileucine; YXXØ, tyrosine-x-x-Ø (where X designates any amino acid and Ø an amino acid with a bulky hydrophobic group).

As clearly observed from the domain structure depicted in Figure 1 and Figure 2, LRP1B is a gigantic LDLR family member closely resembling LRP1. Despite being encoded by different genes (mapped to chromosomes 2 and 12, respectively), LRP1B and LRP1 share 59% cDNA level and 52% amino acid sequence [14] and, most importantly, have a near identical overall structure [1,2,14] (Figure 2). Both these proteins have: i) four extracellular ligand-binding domains (I, II, III, and IV from the N-terminus) that consist of 2, 8, 10, and 12 LDLRA domains, respectively; ii) ligand-binding domains interspaced by three clusters of EGF-like and YWTD β-propeller domains. Similar to LRP1, human LRP1B has a furin cleavage site (REKR, arginine-glutamate-lysine-arginine) between the fourth ligand-binding domain and the transmembrane domain [14], which allows for a furin-mediated proteolytic cleavage event (in the trans-Golgi network) to form the mature receptor as a non-covalently associated heterodimer composed of an N-terminal large subunit (520 kDa α-chain) and a C-terminal smaller subunit (90 kDa β-chain) [14,15,16,17,18,19,20,21]. So far, the physiological significance of this post-translational proteolytic cleavage remains unknown.

Both LRP1 and LRP1B have a cluster of six EGF-like domains adjacent to the transmembrane domain and an intracellular domain with two NPXY, one tyrosine-x-x-Ø (YXXØ, where X designates any amino acid and Ø an amino acid with a bulky hydrophobic group), and two dileucine (LL) motifs [14,22]. Exclusive of LRP1B are (i) an extra LDLRA domain within its fourth ligand-binding domain and (ii) a unique 33 amino acid sequence between the two NPXY motifs of its intracellular domain [14].

### 1.2. LRP1B Function

**Figure 3:**
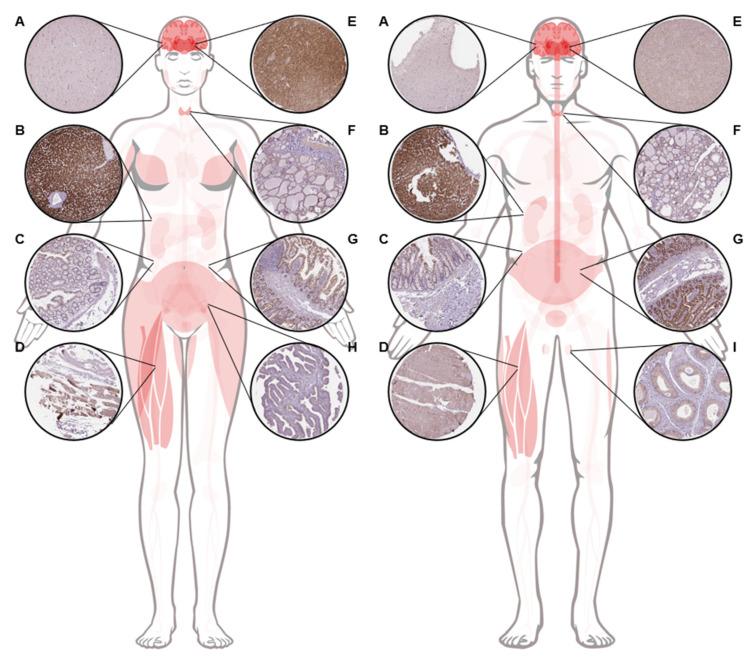
**Anatomogram of LRP1B expression in normal human tissue.** LRP1B expression data was derived from the antibody-based profiling of 76 different cell types corresponding to 44 normal human tissue types. Figure was prepared using anatomograms and immunohistochemistry images from version 20.1 of Human Protein Atlas available from https://www.proteinatlas.org/ENSG00000168702-LRP1B/tissue org (accessed on 14 June 2021) [23,24]. (**A**), cerebral cortex. (**B**), liver. (**C**), colon, (**D**), skeletal muscle. (**E**), caudate. (**F**), thyroid gland. (**G**), small intestine. (**H**), fallopian tube. (**I**), testis.

Human LRP1B is broadly expressed in multiple normal tissues (Figure 3) such as the cerebral cortex, hippocampus, caudate, spinal cord, thyroid gland, adrenal gland, nasopharynx, bronchus, lung, salivary gland, esophagus, stomach, small intestine, colon, rectum, liver, gallbladder, pancreas, skeletal muscle, smooth muscle, soft tissue, adipose tissue, skin, bone marrow, lymph node, testis, prostate, ovary, fallopian tube, cervix, placenta, and breast [3,18,20,23,24].

**Figure 4:**
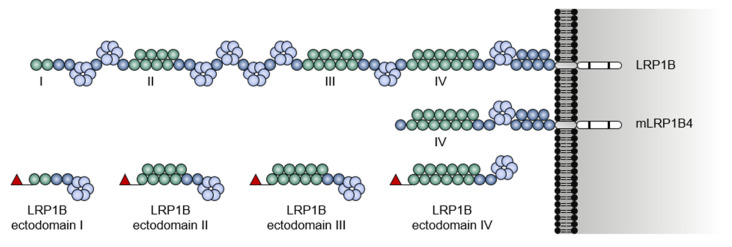
Structure comparison between full-length LRP1B, LRP1B mini-receptor (mLRP1B4), and N-terminal-tagged LRP1B ectodomains I, II, III, and IV. The extracellular ligand-binding domains (I to IV) are identified. The red flag represents the N-terminal tag.

Much of the studies first addressing the function of LRP1B have speculated in-light-of its high structural similarity with LRP1; these pointed out to possible overlapping ligand specificity and functions between these two receptors, some of which have been confirmed [1,14]. Nevertheless, the study on LRP1B exact function and ligands has been hampered by its enormous gene size (approximately 1.90 Mbps long [3]). LRP1B introns are 10 times larger than LRP1 [14]. It is technically challenging to clone its 16.5 kilobases cDNA (with an open reading frame of 13,800 bp) [3,14,21]. This affects the possibility of overexpressing the full-length LRP1B protein (4599 amino acids) in cells. In an attempt to get closer to this hurdle, Beer et al. [21] through a PCR-based cloning strategy, were able to express a full-length murine *Lrp1b* cDNA (13.800 bp) in human tumor cell lines. This contributed to confirm some of the potential roles of LRP1B in cancer (addressed later on in this review). Still, two main approaches have been responsible for the knowledge on potential extracellular ligands of LRP1B. The first, and most commonly described in the literature, uses cellular overexpression of an LRP1B mini-receptor comprising the fourth (IV) ligand-binding domain, transmembrane domain, and intracellular domain (designated mLRP1B4; Figure 4). This allows assessing LRP1B’s ability to bind and internalize ligands after its overexpression in cells [14,17,25,26]. The second approach uses soluble recombinant LRP1B ectodomains containing the first (I), the second (II), the third (III), or the fourth (IV) ligand-binding domains (designated LRP1B ectodomains I, II, III, and IV; Figure 4) to assess their ability to bind various ligands present in mouse brain tissue lysate [18] and human plasma [27].

Together, these approaches provided significant insights into the extracellular ligand specificity and recognition properties of LRP1B (Table 1). These include some already known to bind to the homologous LRP1, including the receptor-associated protein (RAP), the components of the urokinase-type plasminogen activator (uPA) system (i.e., uPA, uPA receptor (uPAR), and plasminogen activator inhibitor-1 (PAI-1)), the tissue-type plasminogen activator (tPA), the amyloid precursor protein (APP), the *Pseudomonas aeruginosa* exotoxin A, and the apolipoprotein E (apoE)-containing lipoproteins HDL and VLDL [28]. Additionally, several well-known chaperones and co-chaperones (sacsin, endoplasmin, DnaJ homolog subfamily A member 1, and clusterin), and other structurally and functionally diverse proteins (synaptotagmin-1, glutathione S-transferase LANCL1, 40S ribosomal protein SA, fibrinogen, histidine-rich glycoprotein, vitronectin, serum amyloid P component, and two immunoglobulin components) were identified as LRP1B ligands (exclusively). These ligands are involved in a wide range of biological processes such as angiogenesis, blood coagulation, fibrinolysis, hemostasis, chemotaxis, cell proliferation, adhesion, spreading, migration, apoptosis, endocytosis, innate and adaptive immunity, host–virus interaction, and protein folding and trafficking [29,30,31,32,33,34,35,36,37,38,39,40,41]. Almost all the identified LRP1B ligands were found to bind to either the second or the fourth ligand-binding domain (Table 1), which interestingly also represent the major ligand-binding sites of LRP1 [28]. Additionally, some ligands were found to bind to more than one ligand-binding domain.

**Figure 5:**
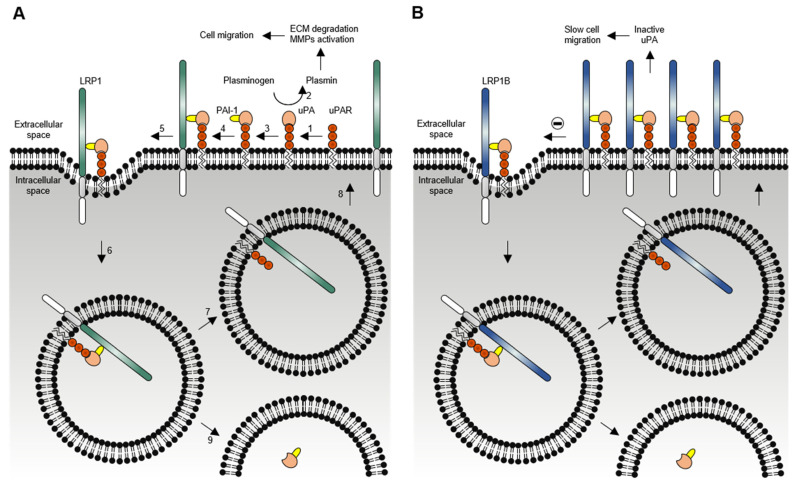
**Model depicting the differential functions of LRP1 (A) and LRP1B (B) in endocytosis of uPAR**. (**A**) uPAR LRP1-mediated endocytosis: (1) uPA binds to uPAR; (2) active uPA catalyzes the conversion of plasminogen to plasmin, which cleaves and activates matrix metalloproteinases (MMPs); both plasmin and MMPs degrade many extracellular matrix (ECM) components; (3) PAI-1 binds to and inhibits uPA; (4) binding of PAI-1 promotes binding of LRP1; (5) fast distribution of the quaternary complexes (uPA/PAI-1/uPAR/LRP1) to clathrin-coated pits; (6) the quaternary complexes are internalized and delivered into early endosomes; (7) sorting of LRP1 and uPAR into recycling vesicles; (8) recycling of unoccupied forms of LRP1 and uPAR back to the cell surface; (9) uPA/PAI-1 complexes are trafficked through late endosomes to lysosomes for degradation. (**B**) Similar to LRP1, LRP1B forms complexes with uPA/PAI-1/uPAR. However, slow endocytosis of LRP1B causes a slow elimination of occupied uPAR from the cell. As a result, occupied uPAR accumulates on the cell surface, functional uPAR is not regenerated effectively, uPA proteolytic activity is scarce, and cell migration is diminished.

**Figure 6:**
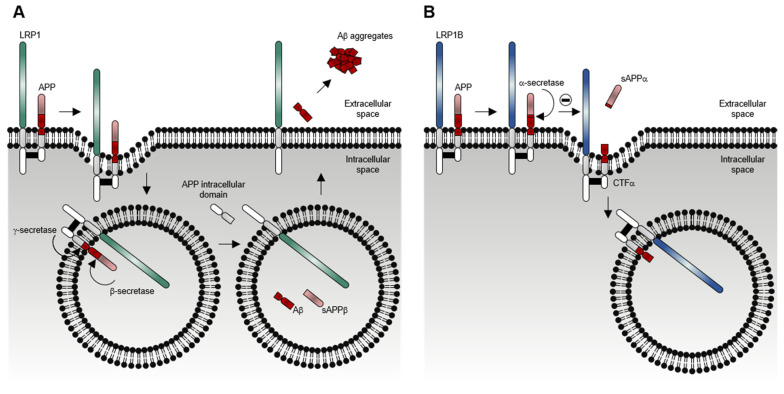
**Model depicting the differential functions of LRP1 (A) and LRP1B (B) in endocytosis of APP.** (**A**) The fast endocytosis of LRP1 enhances APP endocytosis and, therefore, promotes the proteolytic processing of APP through the amyloidogenic pathway. Once delivered to the endosomes, APP is firstly cleaved by a β-secretase, producing a soluble β-cleaved APP fragment (sAPP-β) and a carboxyl-terminal β fragment (CTF-β). This fragment is further cleaved by a γ-secretase, producing the highly toxic amyloid β-peptide (Aβ) and the APP intracellular domain. Most of the Aβ peptides are secreted to the extracellular space, where they can rapidly aggregate and form fibrils that deposit into the amyloid plaques (which are associated with the progression of Alzheimer’s disease). (**B**) The slow endocytosis of LRP1B decreases APP endocytosis and, therefore, promotes the proteolytic processing of APP through the non-amyloidogenic pathway. At the cell surface, APP is firstly cleaved by an α-secretase, producing a soluble α-cleaved APP fragment (sAPP-α) and a carboxyl-terminal α fragment (CTF-α). This fragment can be further cleaved by a γ-secretase, producing the non-toxic peptide P3 and the APP intracellular domain.

Although LRP1 and LRP1B share numerous ligands, the kinetics of receptor-mediated endocytosis for the same ligand may be remarkably different. Using RAP, Liu et al. [14] evaluated its internalization rates by both LRP1 and LRP1B mini-receptors (mLRP4 and mLRP1B4, respectively). mLRP1B4 showed a much slower rate of internalization (t_1/2_ > 10 min) in comparison with mLRP4 (t_1/2_ < 0.5 min). Another study showed that the rate and the extent of uPA/PAI-1 complexes internalization in mLRP1B4-expressing cells was much slower and incomplete than in mLRP4-expressing cells [25]. This study also showed that mLRP1B4, similar to mLRP4, could bind uPA/PAI-1/uPAR complexes and internalize them [25]. By then, it was known that after the internalization of the uPA/PAI-1/uPAR complexes mediated by LRP1, the uPA/PAI-1 complexes were trafficked to lysosomes for degradation, and the unoccupied (ligand-free and active) forms of LRP1 and uPAR were recycled back to the cell surface [42,43,44,45]. However, mLRP1B4-expressing cells, compared with mLRP4-expressing cells, showed a substantially reduced capacity to recycle unoccupied uPAR to the cell surface, which was consistent with the functional difference in the internalization rates of the LRP1 and LRP1B mini-receptors [25], and to migrate. Li et al. showed that LRP1B could act as a negative regulator of uPAR regeneration and cell migration [25]. Later, Cam et al. also showed that mLRP1B4-expressing cells, in contrast to mLRP4-expressing cells, exhibited a considerable accumulation of APP (another of the referred LRP1B extracellular ligands) at the cell surface, in accordance with a low internalization rate of APP mediated by mLRP1B4, and a concomitant decrease in amyloid β-peptide (Aβ) production and increase in soluble α-cleaved APP fragment (sAPP-α) secretion [17]. Models depicting the differential functions of LRP1 and LRP1B in the endocytosis of uPAR and APP are shown in Figure 5 and Figure 6, respectively. Overall, these studies suggest that LRP1B, when expressed in the same cells (or tissues) that LRP1, may antagonize LRP1 function by (i) competing for binding of common ligands and reducing their intracellular catabolism or (ii) modulating the function of other cell-surface receptors.

As for other LDLR family members for which studies have described other roles beside the canonical receptor-mediated endocytosis function, LRP1B is also involved in signal transduction through the interaction of its intracellular domains with cytosolic adaptor and scaffold proteins. To date, eight interacting partners of the LRP1B intracellular domain have been identified (Table 2) which are involved in several biological processes such as signal transduction, synaptic transmission and plasticity, cell migration, tumorigenesis and tumor progression, and DNA damage response [46,47,48,49,50,51,52,53].

One of the best-elucidated interactors is the scaffold protein PICK1 (protein interacting with C kinase 1) [54] which recognizes LRP1B intracellular domain and inhibits its phosphorylation by protein kinase C α (PKC-α). Interestingly, although PICK1 was able to bind to the intracellular domain of LRP1 even more efficiently than to the intracellular domain of LRP1B, PICK1 did not affect LRP1 phosphorylation by PKC-α. A previous study indicated that the phosphorylation of the intracellular domain of LRP1 by PKC-α was responsible for the regulation of the endocytic and signaling activity of this cell-surface receptor by altering its association with its interacting partners [55]. In regard to LRP1B, it appears that the phosphorylation by PKC-α and its regulation by PICK1 modulates the endocytic and signaling activity of LRP1B [54]. Still, further studies are required to comprehend the biological significance of the interactions between LRP1B and its intracellular partners.

**Figure 7:**
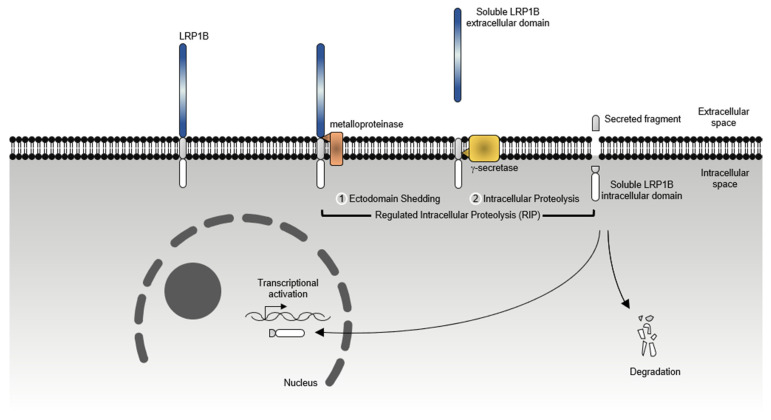
**Regulated intramembrane proteolysis (RIP) of LRP1B.** LRP1B undergoes proteolytic cleavage by a metalloproteinase (i.e., ADAM17) that results in the shedding of its extracellular domain. Then, the remaining 21 kDa membrane-bound carboxyl-terminal fragment undergoes a gamma-dependent intramembrane cleavage that results in the release of the LRP1B intracellular domain (18 kDa) into the cytosol, where it can be degraded or translocated to the nucleus.

Adding to the several LRP1B functions potentiated by the already described extracellular and intracellular ligands, LRP1B function may also be exerted after undergoing regulated intramembrane proteolysis (RIP, a proteolytic process whereby a transmembrane protein undergoes two consecutive proteolytic cleavages by distinct proteases) [56]. Liu et al. [57] showed that LRP1B was first cleaved by a metalloproteinase, leading to the release of its extracellular domain. It was then cleaved within the transmembrane domain by a γ-secretase that led to the liberation of its intracellular domain (Figure 7). This study also demonstrated that LRP1B soluble intracellular domain translocated to the nucleus, via a nuclear localization signal. Nevertheless, the exact function or potential target gene(s) remain unknown [57].

## 2. LRP1B Impairment in Cancer

### 2.1. LRP1B Expression and Roles in Cancer

Knowledge on LRP1B has been closely related to its possible involvement in cancer. In fact, *LRP1B* gene also designated as *LRP-DIT* (LRP-deleted in tumors) was first identified as frequently inactivated in non-small-cell lung cancer cell lines and as a putative tumor suppressor [1]. Since then, several studies have been trying to address the mechanisms involved in LRP1B regulation, as well as the exact role played by LRP1B in cancer [3,21,27,58,59,60,61,62,63,64].

**Figure 8:**
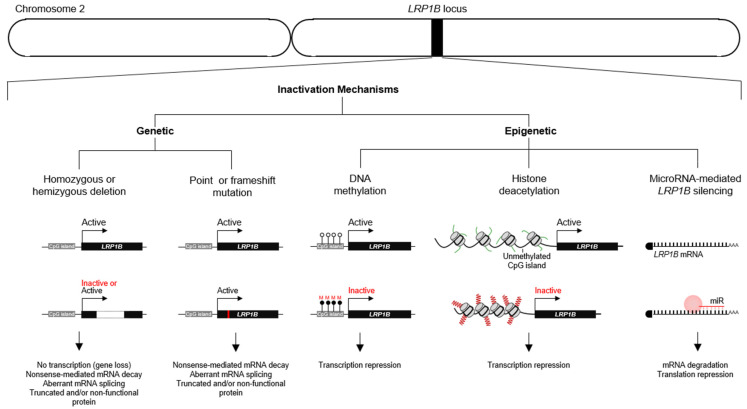
Mechanisms of genetic and epigenetic inactivation of LRP1B in cancer.

LRP1B inactivation is frequent in cancer and known to occur through several different mechanisms at both genetic and epigenetic levels (Figure 8). Beside deletions (homozygous and hemizygous whole- and partial) [1,59,62,64,65,66,67,68,69,70,71,72,73,74,75,76,77,78,79], genetic mechanisms included point mutations (missense, nonsense, and splice-site disrupting) and frameshift mutations (derived from insertions and deletions (indels) [1,79,80,81,82,83,84,85,86,87,88,89,90,91,92,93,94,95,96].

Moreover, epigenetic mechanisms are known to affect *LRP1B* expression in several cancers; these include hypermethylation of CpG island located in the *LRP1B* promoter region [62,64,68,70,78,97,98,99,100], histone deacetylation of the *LRP1B* promoter region [78], and microRNA (miRNA)-mediated post-transcriptional regulation such as miR-548a-5p [thyroid cancer [62], miR-500 and miR-301b-3p, and prostate cancer [101,102]. Interestingly, *LRP1B* is also described as a common target gene for viral integration. This has been observed for hepatitis B virus (HBV) in liver cancer [103] and for human papillomavirus (HPV), resulting in LRP1B-downregulated expression in cervical cancers [104].

Over the years it was possible to confirm that *LRP1B* features the group of most altered genes across human cancers [21,59,62,65,68,69,78,97]. Recently, based on data from “The Cancer Genome Atlas TCGA” available on the cBioPortal database, Brown et al. showed that *LRP1B* somatic alterations were identified in approximately 12% across all samples and in more than 20% in multiples tumor types including NSCLC, melanoma, esophageal, stomach, head and neck, uterine, and bladder cancers [58]. Within the most common genetic alterations are LRP1B mutation, LRP1B loss, LRP1B R2553Q, LRP1B S2508F, and LRP1B E4182K (0.18%) [105].

Despite the frequency of LRP1B dysfunction in cancer overall, there is still a lack of information on how this affects its expression and/or functional role. LRP1B significance as a potential therapeutic target or biomarker for cancer prognosis or response to therapy remains to be fully elucidated [60,63,89].

Regardless of the existing limitations (already referred) to study the impact of LRP1B expression in tumor cells, several studies using tumor cell lines (and some using their xenografts in nude mice) have contributed to the knowledge on the role of LRP1B in cancer known so far.

Through the re-establishment of LRP1B expression (with mini-receptor) in LRP1B-deficient cancer cells, it was observed the suppression of anchorage-dependent growth (in esophageal [68] and gastric cancer cells [100]) and anchorage-independent growth (brain [57], gastric [100], thyroid [62] and colon cancer cells [106]). Interestingly, it was also found that cells transfected with a mutant mLRP1B4 (resistant to the extracellular domain release) were less able to suppress colony formation. To further determine whether this observation was linked to the regulated intramembrane proteolysis of LRP1B, the authors performed a soft agar assay with cells transfected with the intracellular domain of LRP1B, and discovered that the intracellular domain of LRP1B per se was able to suppress anchorage-independent growth similar to mLRP1B4 [57], suggesting that LRP1B tumor suppressor activity requires its proteolytic processing. Still, further studies are needed to identify target genes of the intracellular domain of LRP1B. This may provide other insights into its specific role.

Moreover, the restoration of LRP1B expression with the mini-receptor: (i) reduced cell proliferation in gastric cancer cell lines and tumorigenicity in nude mice [100]; (ii) suppressed cell proliferation in ovarian cancer cell lines [59,60]; (iii) inhibited cell proliferation and migration in colon cancer cell lines [106], and (iv) decreased in vivo tumor development and growth, impaired cell invasion, and modulated thyroid cancer cells’ secretome [62]. LRP1B (mini-receptor) expression in thyroid cells altered their secretome, particularly by reducing the levels of MMP-2 (involved in the degradation of ECM). This further contributes to LRP1B endocytic receptor role as a tumor suppressor through modulation of the tumor microenvironment.

On the other hand, LRP1B-silencing with small interfering or short hairpin RNAs (siRNAs or shRNA, respectively) resulted in: (i) increased anchorage-independent growth, cell migration, and invasion in renal cancer cell lines [78], (ii) enhanced cell proliferation in lung cancer cell lines [21], and (iii) promoted anchorage-independent growth, cell proliferation, and migration in colon cancer cell lines [106]. Interestingly, Ni et al. found that the increased migration and invasion of LRP1B-silenced renal cancer cells was possibly due to actin cytoskeleton remodeling regulated by the Rho/Cdc42 pathway, and the alteration of focal adhesions complex components [78]. Zhang et al. showed that miR-500 promoted cell proliferation by directly targeting LRP1B mRNA in prostate cancer cells [101]. Zheng et al. also showed that the up-regulation of miR-301b-3p (induced by hypoxia) promoted cell proliferation, migration, and invasion of prostate cancer cells and enhanced tumorigenicity in nude mice through negative regulation of LRP1B expression [102].

### 2.2. LRP1B as a Therapeutic Target, Prognostic Factor, and Predictor of Response to Therapy

Most recently, increasing knowledge on LRP1B is pushing towards its relevance cancer, not only as a tumor suppressor gene but further exploring its potential as a target or as a putative translational biomarker. Adding on to what is already known, the advance in methodologies that better allow to manipulate LRP1B expression in cells (such as CRISPR-cas) and to more thoroughly explore its expression and mutation status (such as with new available antibodies and dramatic improvement in genomic analysis) will most definitely contribute to bring new light on the role of LRP1B. 

So far, Guo et al. used both CRISPR-Cas9 silencing and CRISPRa transcription activation systems to, respectively, downregulate and upregulate LRP1B expression in hepatocellular carcinoma cells (HCC). In this study, in contrast to what is mostly reported for cancer cells, LRP1B expression is predominantly strong [107]. This study presented a unique role for LRP1B in this setting, as a mediator in lipid metabolism, and further proposes LRP1B-mediated lipid metabolism as a potential therapeutic strategy for HCC in the future [107].

LRP1B expression and intracellular localization are also being considered regarding their role as prognosis and/or predictive response biomarkers.

Using antibodies for LRP1B intracellular domain, Asano et al. found not only a membrane and cytoplasmic LRP1B expression pattern in the majority of the invasive ductal breast carcinoma patient samples analyzed, but also nuclear expression in a lower number of cases. Importantly, nuclear LRP1B staining was shown to significantly associate with poor patient prognosis, in particular with luminal A-type breast cancer [108].

More recently, evaluation of LRP1B expression in gastric cancer patient samples showed that cytoplasmic LRP1B was significantly associated with a low clinicopathological stage and favorable prognosis of patients with diffuse-type gastric cancer, but not with intestinal-type gastric cancer [109]. Cytoplasmic LRP1B was considered an independent favorable prognostic value in diffuse-type gastric cancer [109].

LRP1B expression has also been associated with cancer response to therapy. Being a mediator of the clearance of various extracellular ligands from the cellular environment, such as liposomes (often used to encapsulate chemotherapeutic drugs), LRP1B endocytic activity may have clinical impact (affecting drug uptake). Its dysfunction may therefore contribute to resistance to liposomal therapies, namely to pegylated liposomal doxorubicin-PLD [59,60,63]. Cowin et al. presented LRP1B loss (analyzed at DNA and mRNA levels) as potential contributor to the emergence of resistance to liposomal doxorubicin in high-grade serous ovarian cancer patients [59]. Functional studies in ovarian cancer cell lines showed that LRP1B overexpression (with a mini-receptor) increased their sensitivity to liposomal doxorubicin [59,60], while its downregulation had the opposite effect [59]. More recently, the expression of LRP1B was evaluated for the first time at protein level in a series of ovarian cancer patients. In this study, higher LRP1B levels associated with prolonged patient progression-free-survival (PFS), being more evident for PLD treated patients [63]. This further supports the role of LRP1B as putative predictor of response to PLD [59,60,63].

Driven by the advances on genomics analysis and consequent application of whole-exome and NGS sequencing platforms in clinical oncology [110], exhaustive sequencing of all *LRP1B* gene is now possible [111]. *LRP1B* genetic landscape analysis should therefore no longer be ignored, as pointed out by the results published. Recent studies are disclosing the impact of *LRP1B* gene alterations in cancer patient prognosis as well as in their sensitivity to immune checkpoint inhibitors (ICI) in multiple tumors [58,61,79,89,112,113]

For example, the *LRP1B* mutation rate was found to be high in HCC patients and to correlate with worse prognosis [61]. In this setting, it was described as an independent risk factor affecting patients’ prognosis [61] and a model for patients’ risk prediction, based in LRP1B mutation, has been developed and validated [112]. In the past, LRP1B alterations (such as deletions evaluated by SNP-array) were found to associate with glioblastoma patients’ poor prognosis [64].

Immune checkpoint inhibitors (ICIs), such as anti- PD-(L)1 and anti-CTLA-4, have been gaining momentum by considerably improving outcomes in several cancers [58,114,115]. Consequently, the number of immunotherapy predictors described has been raising over the years, among which are included tumor mutation burden (TMB, a measurement of the number of mutations harbored by tumor cells), expression of immune checkpoint genes, microsatellite instability, and DNA mismatch repair defects [61,116,117,118]. Recently, *LRP1B* mutation status was associated with improved patients’ outcome to immunotherapy, being described as a biomarker for ICIs in multiple cancers such as melanoma, non-small cell lung cancer, prostate cancer, and advanced biliary tract cancer [58,79,89,119]. However, the exact mechanisms involved warrant further study.

Among the most often described associations of *LRP1B* mutations is TMB (per se, an independent indicator to stratify patients’ sensitivity to immunotherapy). *LRP1B* has been described as a predictive biomarker of TMB [89,92,114,117]. *LRP1B* large size and its location near the FRA2F fragile site may contribute toward higher TMB [58]. TMB is usually evaluated using expensive, massive next-generation, or whole-exome sequencing [120]. Nevertheless, specific single-gene mutation analysis has been presenting as a more accessible way to predict TMB in cancer patients [120], with *LRP1B* being one of those genes [61,120,121]. In HCC, *LRP1B* mutation significantly associated with a higher HHLA2 (human endogenous retrovirus-H long terminal repeat-associating protein 2), as well as with TMB, giving new insights into potential targets for immune checkpoint inhibition [61]. Several questions remain on whether the LRP1B functional role depends on other factors and/or if it may act independently. A study by Jonhson et al. in melanoma patients treated with ICI (anti-PD1) showed that *LRP1B* mutations were significantly enriched in the responder group (34%) when compared with the non-responder group (3%). Moreover, the study showed that there was an increase in TMB in *LRP1B*-mutated patients in relation to those of the *LRP1B* wild-type [58,111]. Additionally, in melanoma and NSCL patients, *LRP1B* mutations significantly associated with a better ICI survival outcome, even after excluding the potential effect of other factors, such as TMB.

A large multicenter, retrospective study by Brown et al. showed an association of *LRP1B* mutation with favorable outcome to ICIs (anti-PD-(L1)1 and anti-CTLA-4) across several cancer types. In this study, LRP1B mutations were defined in three groups: pathogenic (leading to large deletion, truncation or loss of function); likely pathogenic (missense mutations listed in COSMIC database scored for likelihood of pathogenicity of >0.5 with Functional Analysis through Hidden Markov Models—FATHM), or variants of unknown significance (VUS, missense alterations not listed in COSMIC, or with FATHMM scores of <0.5). Authors reported significantly better outcomes to ICI therapy (including improved response rates, progression-free survival, and overall survival) in patients whose tumors had pathogenic (or likely pathogenic) LRP1B alterations, compared with those with LRP1B VUS [58]. Despite the fact that this study has classified some LRP1B alterations as “pathogenic (like)”, the true effect of specific mutations on LRP1B functional role(s) both in physiologic as in pathologic conditions needs to be evaluated and might be better understood over time [58].

Different studies have been proposing other potential mechanisms for the impact of LRP1B mutations in immune response. In HCC patients bearing *LRP1B* mutations, differential expression analysis showed increased expression of several DNA repair mechanisms (base excision repair, nucleotide excision repair, and mismatch repair) [61]. Moreover, in melanoma and NSCL patients, cell cycle regulation and antigen processing and presentation pathways were significantly altered in samples from patients bearing LRP1B mutations [89]. Additionally, as infiltration of immune cells in the tumor microenvironment is known to impact immunotherapy, some authors have been evaluating its relation to *LRP1B* mutations. A study by Bao et al. described *LRP1B* as the fifth most commonly mutated gene in T cell-inflamed tumors, despite losing significance when adjusted for TMB [122]. In melanoma and NSCL patients, *LRP1B*-mutated patients were shown to present enriched antigen presentation and interferon-related circuits as well as higher T cell-inflamed gene expression [89]. Evaluation of tumor-infiltrating lymphocyte cells in a melanoma microenvironment showed an enrichment of CD8^+^ T cells, activated CD4 memory T cells, activated NK cells in the *LRP1B* mutant type group, while neutrophils were enriched in the wild-type group [89]. Likewise, in HCC patients the infiltration of naive CD4 T cells was significantly higher, and of neutrophils significantly lower, in the LRP1B mutant group compared with the wild type group [61].

## 3. Conclusions

Despite *LRP1B* being among the most altered genes in human cancers, and the studies throughout the years supporting its role in cancer, there are still several missing links which do not allow to fully translate its relevance to the clinics. With the development of new research approaches, it is now becoming increasingly evident that further studies on this giant molecule will bring new opportunities for its use as a potential therapeutic target or cancer biomarker for prognosis or prediction of response to therapy.

## Figures and Tables

**Table 1:** Extracellular ligands and membrane-associated receptors of LRP1B.

Ligand		Description	Ligand-Binding Domain (I to IV)	Reference
RAP		Chaperone	II, IV	[14,18]
uPA		Serine protease	IV	[14]
tPA		Serine protease	IV	[14]
PAI-1		Serine protease inhibitor	IV	[14]
uPAR		Cell-surface receptor	IV	[25]
APP		Cell-surface receptor	IV	[17]
Sacsin		Co-chaperone	IV	[18]
Endoplasmin		Chaperone	II, III	[18]
Synaptotagmin-1		Calcium ion sensor	IV	[18]
DnaJ homolog subfamily A member 1		Co-chaperone	II	[18]
Glutathione S-transferase LANCL1		Transferase	IV	[18]
40S ribosomal protein SA		Host cell receptor for virus entry, cell-surface receptor for laminin, ribonucleoprotein	II, IV	[18]
*Pseudomonas aeruginosa* exotoxin A		*P. aeruginosa* toxin	IV	[26]
Fibrinogen	α-chain	Substrate for thrombin, plasmin, and fibrin stabilizing factor	II	[27]
	β-chain		IV	[27]
	γ-chain		IV	[27]
HRG		Plasma glycoprotein	II	[27]
Clusterin		Chaperone	II	[27]
Vitronectin		Glycoprotein found in blood and the extracellular matrix	II	[27]
SAP		Plasma protein	II	[27]
IGKV 1-5		Variable domain of immunoglobulin light chains	II	[27]
IGHA1		Constant region of immunoglobulin heavy chains	II	[27]
HDL		apoE-containing lipoprotein	II	[27]
VLDL		apoE-containing lipoprotein	II, IV	[27]

Abbreviations: RAP, receptor-associated protein; uPA, urokinase-type plasminogen activator; tPA, tissue-type plasminogen activator; PAI-1, plasminogen activator inhibitor-1; uPAR, urokinase plasminogen activator surface receptor; APP, amyloid precursor protein; LanCL1, bacterial lantibiotic synthetase component C-like 1; HRG, histidine-rich glycoprotein; SAP, serum amyloid P component; IGKV 1-5, immunoglobulin kappa variable 1-5; IGHA1, immunoglobulin heavy constant alpha 1; HDL, high-density lipoprotein; VLDL, very-low-density lipoprotein; apoE, apolipoprotein E.

**Table 2:** Interacting partners of the intracellular domain of LRP1B.

Intracellular Interacting Partner	Description	Reference
PSD-95	Scaffold protein	[18]
AIP	Co-chaperone	[18]
JIP-1b	Scaffold protein	[54]
JIP-2	Scaffold protein	[54]
PICK1	Scaffold protein	[54]
RanBP9	Scaffold protein and adaptor protein	[54]
GRB7	Adaptor protein	[54]
G2SNT	Adaptor protein	[54]

Abbreviations: PSD-95, postsynaptic density protein 95; AIP, aryl hydrocarbon receptor-interacting protein; JIP-1b, C-Jun N-terminal kinase (JNK)-interacting protein 1b; JIP-2, C-Jun N-terminal kinase (JNK)-interacting protein 2; PICK1, protein interacting with C kinase 1; RanBP9, ran-binding protein 9; GRB7, growth factor receptor-bound protein 7; G2SNT, gamma-2-syntrophin.

## Data Availability

Data sharing not applicable.
